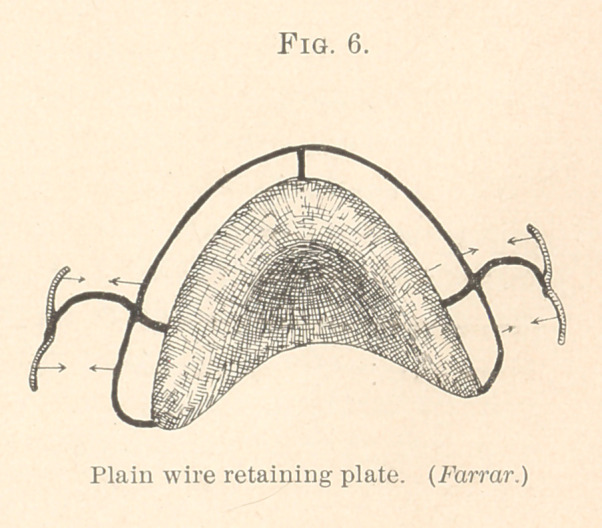# The Scallop Wire Plate for Retaining Teeth in Place after Widening the Dental Arch, and for Correcting Slight Irregularities When Necessary

**Published:** 1894-03

**Authors:** J. N. Farrar

**Affiliations:** New York City


THE
### International Dental Journal.

Vol. XV. March, 1894. No. 3.

### Original Communications.[1]

### THE SCALLOP WIRE PLATE FOR RETAINING TEETH IN PLACE AFTER WIDENING THE DENTAL ARCH, AND FOR CORRECTING SLIGHT IRREGULARITIES WHEN NECESSARY.

BY J. N. FARRAR, M.D., D.D.S., NEW YORK CITY.

To devise regulating mechanisms that will cause force, approximating in accuracy that derived from the screw, without using the screw, and to devise retaining mechanisms that will do no harm to the teeth, has been one of my aims, until I now have a considerable number perfected. In some of these the plate constitutes a part, and in others it does not. In this paper I shall confine myself to the former, leaving some of the other class for future presentation.

After widening or enlarging a dental arch, the teeth should be retained in place artificially for about a year, sometimes longer. For this purpose, roof-plates for the upper jaw and U-shape alveolar ridge-plates for the lower are generally used. If these plates are put in the mouths of careless patients who do not keep their teeth clean there is danger of causing injury to the teeth where the margin of the plate rests against the enamel. Of all retaining-plate mechanisms that I ever devised or ever have seen for such

[1] The editor and publishers are not responsible for the views of authors of papers published in this department, nor for any claim to novelty, or otherwise, that may be made by them. No papers will be received for this department that have appeared in any other journal published in the country.

cases, I think the scallop wire plate is the best, because it fills the office for which it is intended and permits no food *débris* to remain sufficiently long to do injury.

In Fig. 1, which represents one of these mechanisms for an upper arch, W indicates the scallop wire and R the hard rubber plate. This plate fits the roof of the mouth, but does *not* extend so far as to be in contact with the teeth. *There is a space about one-eighth of an inch between the plate and the scallop wire.* This wire, which is bent in zigzag form to fit the lingual surfaces of the teeth, is anchored to the plate by three wire arms, P, P, P, one end of each of which is soldered to the scallop wire, the other embedded in the substance of the plate at the time it is vulcanized.

For the upper jaw there are two ways of holding the mechanism on the teeth; one by an air-chamber, the other by Tomes's clasps, a modification of the Schange crib (1848). In rare instances, however, both are useful. The Tomes clasp does not encircle teeth, but extends over them, so to speak, the wire resting along the *approximal valley* on the grinding-surfaces, thence curving towards the gum on the buccal side, where it grips one or two teeth near their necks. The scallop wire, the clasps, and the arms are made of round wire about the size of a small knitting-needle, each clasp being in union with one of the posts.

It may be thought that the clasps will move inward the teeth borne upon; this, however, would be erroneous, because the clasp and the scallop wire on the opposite side of the tooth holds it firmly, as if in a vise.

Fig. 2 represents a scallop wire retaining and regulating plate, having two clasps on the sides and a double hook in front. The latter was for holding the central incisors in place after having been turned during the time the arch was being enlarged.

This plate has advantages other than that of safety. One of the best of these is, that it can be made to correct irregularities when one or more of the teeth have not been moved sufficiently far, or when teeth have lost ground and again become irregular. Such can be pushed into line by repeatedly bending outward the part of the wire in contact with the tooth or teeth to be moved so that it will press against them. The office of this plate, when first made, was to retain all the teeth in place except the cuspids, which required to be moved still farther outward, and which were corrected by the mechanism.

*Smaller Scallop Wire Plates.*—Smaller plate mechanisms, made on the same principle for holding a less number of teeth (even one

Fig 1.

Upper scallop wire retaining plate. (*Farrar.*)

Fig. 2.

Upper scallop wire retaining and regulating plate. (*Farrar.*)

Fig. 3.

Scallop wire plate for regulating the upper bicuspids and then retaining them in line. (*Farrar.*)

Fig. 4.

Lower scallop wire retaining plate. (*Farrar.*)

Fig. 5.

Outside scallop wire retaining plate. (*Farrar.*)

Fig. 6.

Plain wire retaining plate. (*Farrar.*)

or two) in place, are very practicable. Such partial scallop plates are also useful for moving outward instanding teeth.

Fig. 3 represents a mechanism used for moving outwardly four instanding bicuspids, and then holding them in line. (See Fig. 4.)

*For the Lower Arch.*—A scallop wire plate for the *lower* arch is made U-shape, but otherwise it is the same as that for the upper.

Upon a similar principle I sometimes construct scallop wire mechanisms for moving *outstanding* teeth to line. This is done by having the wire bear upon the outer surfaces of the teeth. For most cases, however, where only one or two are outstanding, I prefer the Tomes clasp to scallop wire. Such clasps are made exactly like those represented in Figs. 1 and 2. Having cut a small part of the plate away on the lingual side of the tooth, the clasp is rebent every day or two against the tooth, thus maintaining the pressure until the tooth is moved into line. The same motion of a tooth can be accomplished when a scallop wire plate is used. Instead of cutting away a part of the plate, the scallop behind the tooth to be moved is bent inwardly. (See Fig. 5.)

When it is difficult to cause a scallop of the wire to bear hard against a cuspid or incisor, because of the inclination of the lingual side of the tooth, it becomes necessary to cement a ferule on it. When applying the mechanism, the scallop wire should rest on the teeth sufficiently high to serve its object, but not so far as to injure the gum. When the wire causes a red line along the gingival margin of the gum, it should be bent away from it.

To bend a scallop, I sometimes apply a chisel between the edge of the plate and the wire, and then turn it, thus forcing the wire farther from the plate. Generally, however, I prefer to use pincers, with beaks broader than the curve of the wire. The wire should not be so soft that it will not remain in proper form when tightly forced within the arch; nor so hard that it will spring out of proper shape in one place when a section elsewhere is being altered.

The wire for these mechanisms may be of gold or German silver; I prefer the former. In constructing the mechanism the wire is first bent by means of short, round, beaked pincers, until it fits accurately the curved surfaces of the lingual side of each tooth. It is then removed from the cast, and the arms soldered to it at places most desirable. After this, all is replaced on the cast, and the arms bent so that they will lie near to the alveolar surface, when they are ready to have the rubber plate vulcanized to them. When vulcanized the rubber is cut away one-eighth of an inch from the wire.

*Plain Wire Retaining Plate.*—I sometimes make plates on the plan of Fig. 1, except the scalloping of the wire, the latter being left straight or plain all the way around the arch. While this mechanism will retain a widened or enlarged arch in proper form, it will not prevent sidewise movements of the teeth, if they are disposed to so move; nor does the plain wire permit of alteration of form in one place without causing a change of form elsewhere; it is therefore comparatively useless for regulating teeth. (See Fig. 6.)

## Figures and Tables

**Fig 1. f1:**
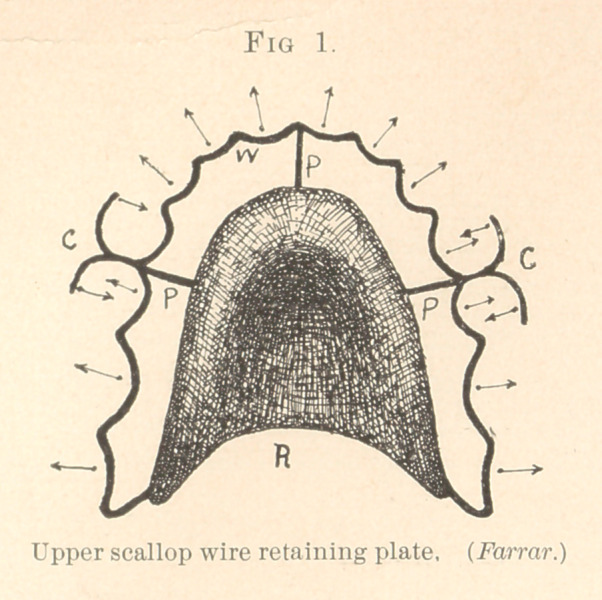


**Fig. 2. f2:**
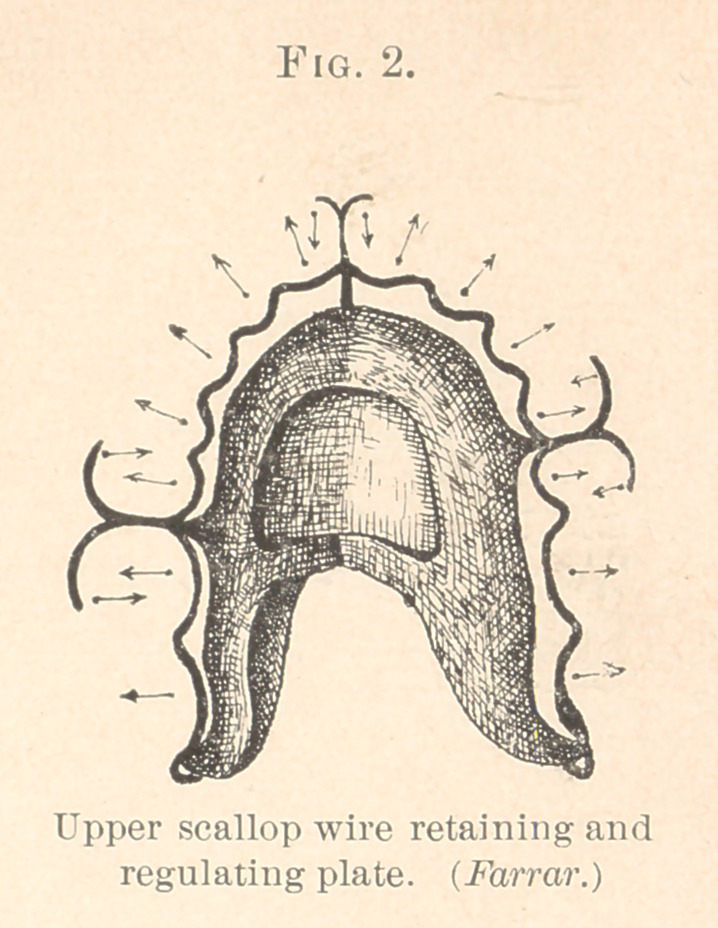


**Fig. 3. f3:**
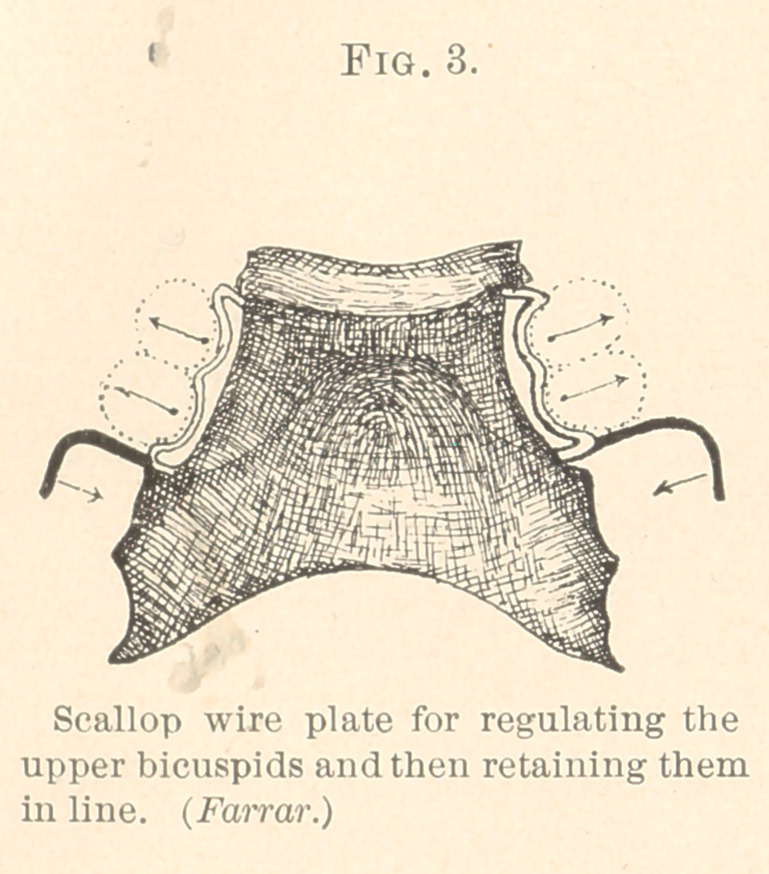


**Fig. 4. f4:**
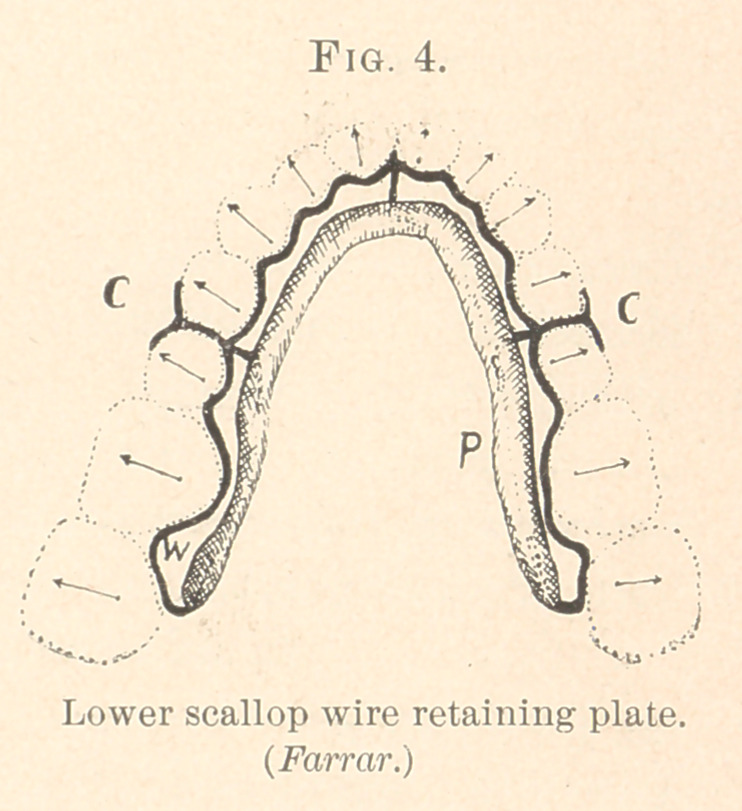


**Fig. 5. f5:**
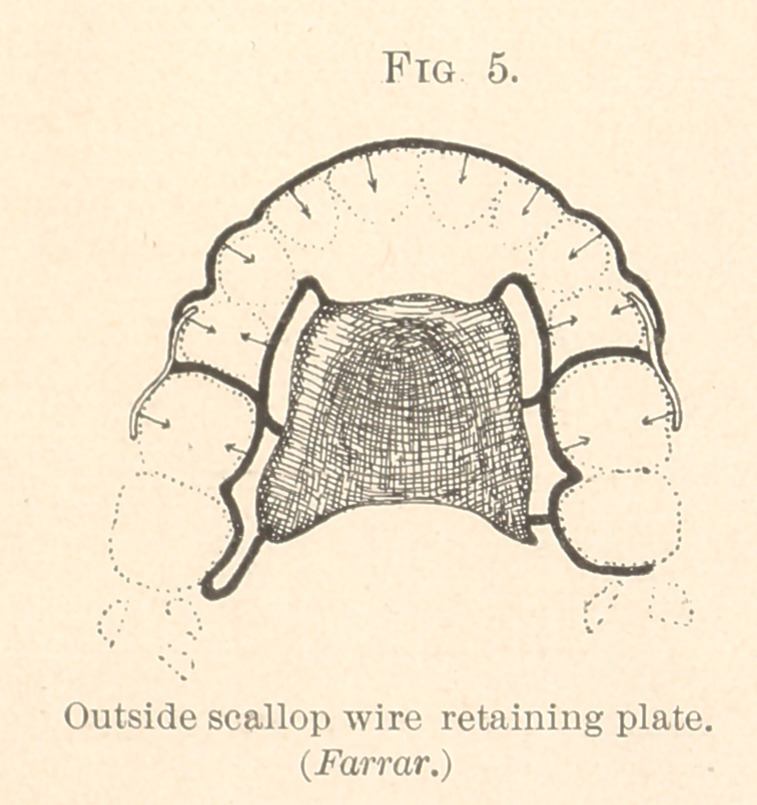


**Fig. 6. f6:**